# Weiterentwicklung und Inhaltsvalidierung eines Fragebogens zur Erfassung des Wissens über das Delir

**DOI:** 10.1007/s00391-022-02015-9

**Published:** 2022-01-26

**Authors:** Max Zilezinski, Renée Lohrmann, Armin Hauß, Manuela Bergjan

**Affiliations:** 1 Charité – Universitätsmedizin Berlin, corporate member of Freie Universität Berlin, Humboldt-Universität zu Berlin, and Berlin Institute of Health, Geschäftsbereich Pflegedirektion – Pflegewissenschaft, Charité – Universitätsmedizin Berlin, Charitéplatz 1, 10117 Berlin, Deutschland; 2 Universitätsmedizin Halle (Saale), AG Versorgungsforschung | Pflege im Krankenhaus, Department für Innere Medizin, Medizinische Fakultät, Martin-Luther-Universität Halle-Wittenberg, Ernst-Grube-Str. 40, 06120 Halle (Saale), Deutschland; 3 Medizinische Fakultät, Martin-Luther-Universität Halle-Wittenberg, Dorothea Erxleben Lernzentrum Halle (DELH), Projekt FORMAT, Magdeburger Straße 12, 06112 Halle (Saale), Deutschland

**Keywords:** Delir, Evaluation, Edukation, Pflege, Content Validity Index, Delirium, Evaluation, Education, Nursing, Content validity index

## Abstract

**Hintergrund:**

Das Delir ist ein neuropsychiatrisches Syndrom, welches häufig ältere Patient_innen betrifft und schwerwiegende Folgen haben kann. Oftmals wird es vom Gesundheitspersonal nicht erkannt. Der Wissensstand über das Delir ist beim pflegerischen und ärztlichen Personal häufig unzureichend ausgeprägt. Zum aktuellen Zeitpunkt fehlt im deutschsprachigen Raum ein Fragebogen zur Erfassung des Wissensstands.

**Ziel:**

Weiterentwicklung eines Fragenbogens und Bewertung der Inhaltsvalidität.

**Methode:**

Im Rahmen einer Literaturrecherche wurden mehrere Fragebögen identifiziert. Ein bereits publizierter Fragebogen mit den Dimensionen Grundlagenwissen über das Delir und Risikofaktoren ist übersetzt, angepasst und um die Dimension der nichtpharmakologischen Delirprävention erweitert worden. Die Bewertung der Fragebogenitems erfolgte durch Delirexpert_innen in 2 Runden. Die Inhaltsvalidität wurde anhand des Content Validity Index (CVI) auf Item(I-CVI)- und Skalen(S-CVI)-Level angegeben, zusätzlich wurde der „modified Kappa“ (κ*) mit der Untergrenze des 95 %igen Konfidenzintervalls (KI) berechnet.

**Ergebnisse:**

Der 30 Items umfassende Originalfragebogen wurde um 18 Items der Delirprävention erweitert. Nach der ersten Bewertungsrunde durch 13 Expert_innen zeigten 30 von 48 Items gute bis exzellente I‑CVI-Werte (0,78–1,0). Unter Berücksichtigung der Kommentare wurden 6 Items verworfen und 12 Items sprachlich und inhaltlich adaptiert. In der finalen Version des Fragebogens verblieben 41 Items mit exzellenten Werten (1,0). Der Gesamtskalenwert hatte sich von 0,88 in der ersten Version auf 1,0 in der finalen Version erhöht. Als Zielgruppe wurden Pflegefachpersonen identifiziert, aber potenziell auch therapeutisches und ärztliches Personal.

**Schlussfolgerung:**

Der Fragebogen zur Erfassung des Wissens über das Delir ist inhaltsvalide.

**Zusatzmaterial online:**

Zusätzliche Informationen sind in der Online-Version dieses Artikels (10.1007/s00391-022-02015-9) enthalten.

## Abstract (en)

**Background:**

Delirium is a neuropsychiatric syndrome that can have serious consequences and is often overlooked by healthcare professionals. The level of knowledge about delirium is often insufficient among nursing and medical staff. At the current time there is no suitable questionnaire to record the level of knowledge in German-speaking countries.

**Aim:**

Development of a questionnaire and evaluation of content validity.

**Methods:**

Following a literature search to identify current best practice, several questionnaires were identified. An already published questionnaire with the dimensions of basic knowledge of delirium and risk factors has been translated, adapted and extended by the dimension of nonpharmacological delirium prevention. Delirium experts assessed the relevance of the questionnaire items in two rounds of reviews. Content validity was calculated using the Content Validity Index (CVI) at item (I-CVI) and scale (S-CVI) level. Additionally, the modified Kappa (k*) was calculated using a lower 95% confidence interval (CI).

**Results:**

The original 30-item questionnaire was expanded to include 18 delirium prevention items. After the first round of scoring 30 out of 48 items showed good to excellent I‑CVI scores. Considering the comments, 6 items were discarded and 12 were adapted in terms of language and content. In the final version of the questionnaire 41 items with excellent scores remained. The total scale score increased from 0.88 in the first version to 1.0 in the final version. Nurses were identified as the target group, potentially also therapists and medical personnel.

**Conclusion:**

The delirium knowledge questionnaire is content-valid.

Das Delir kann für betroffene Patient_innen schwerwiegende Folgen haben. Ältere Patient_innen gehören zur höchsten Risikogruppe für die Entwicklung eines Delirs. Relevante Risikofaktoren und die Wirkung nichtpharmakologischer Präventionsmaßnahmen sind vielfach in Studien belegt worden. Der Wissensstand über das Delir ist jedoch beim Gesundheitspersonal oft unzureichend ausgeprägt. Um das Wissen über das Delir zu evaluieren und Schulungen bedarfsgerecht adressieren zu können, wurde erstmals ein deutschsprachiger Fragebogen entwickelt und auf seine Inhaltsvalidität hin überprüft.

## Hintergrund

Das Delir ist eine häufig auftretende akute Störung der Aufmerksamkeit, des Bewusstseins und der kognitiven Fähigkeiten, v. a. bei Patient_innen über 65 Jahren im Krankenhaus. Das Delir stellt ein ernst zu nehmendes Krankheitsbild dar, welches schwerwiegende und langfristige Folgen haben kann [18, 25]. Neben der starken emotionalen Belastung für die Betroffenen und das behandelnde Team zählen u. a. eine erhöhte Morbidität, eine langfristige kognitive Beeinträchtigung sowie ein erhöhtes Risiko für eine Verlegung in eine Pflegeeinrichtung dazu [18, 25]. Die Mortalität bei älteren Patient_innen mit einem Delir gegenüber älteren Patient_innen ohne ein Delir ist über das Dreifache (Odds Ratio 3,18 [95 %-KI: 2,73, 3,70]) erhöht [2]. Die Delirhäufigkeit für ältere Patient_innen auf Allgemeinstationen wird mit 29–64 % angegeben, auf Intensivstationen mit 20–80 % [10, 18]. Dass das Delir potenziell in bis zu 40 % aller Fälle vermeidbar ist, wurde durch die Anwendung nichtpharmakologischer Präventionsmaßnahmen in multidisziplinären Multikomponentenprogrammen nachgewiesen [15, 16, 18]. Das Delir wird jedoch in bis zu zwei Dritteln aller Fälle vom Gesundheitspersonal (Pflegefachpersonen und Mediziner_innen) nicht erkannt und demzufolge nicht behandelt [6, 7, 17]. Als Gründe für die unzureichende Identifikation eines Delirs werden Wissensdefizite und die fehlende routinemäßige Anwendung verlässlicher Delirscreening- und Assessmentinstrumente diskutiert [19, 23]; dies wird in internationalen Leitlinien bei Patient_innen mit Delirrisiko empfohlen. Die Edukation des Gesundheitspersonals wird ebenfalls adressiert [24, 29].

Laut Christensen et al. [5] haben 69 % des Pflegefachpersonals keine Schulung zum Thema Delir erhalten bzw. haben einen moderaten bis unzureichenden Wissensstand zum Delir [8, 22]. Vorrangig fehlt es an Wissen über das Erkennen eines Delirs, Risikofaktoren für eine Delirentwicklung sowie allgemein zum Delirmanagement [8, 13, 16, 22, 30]. Wissensdefizite und eine unzureichende Anwendung von Assessmentinstrumenten werden auch kritisch für Mediziner_innen diskutiert [19, 23]. In der internationalen Literatur gibt es eine überschaubare Anzahl an Fragebögen, die neben dem Wissen hauptsächlich die Einstellung, Haltung und das Selbstvertrauen in das eigene Handeln beim Delirmanagement erfragen. Der Fokus dieser Studie liegt in der Entwicklung eines ersten deutschsprachigen Fragebogens zur Erfassung des Wissens über das Delir.

Ziel des vorliegenden Beitrags ist es, den Prozess der Auswahl, Übersetzung und Weiterentwicklung des Fragebogens sowie die Bewertung der Inhaltsvalidität zu beschreiben. Ein übergeordnetes Ziel war es, ein interprofessionell entwickeltes Edukationsprogramm zu evaluieren und noch bestehende Wissenslücken zielgerichtet zu adressieren.

## Methode

**Figure 1:**
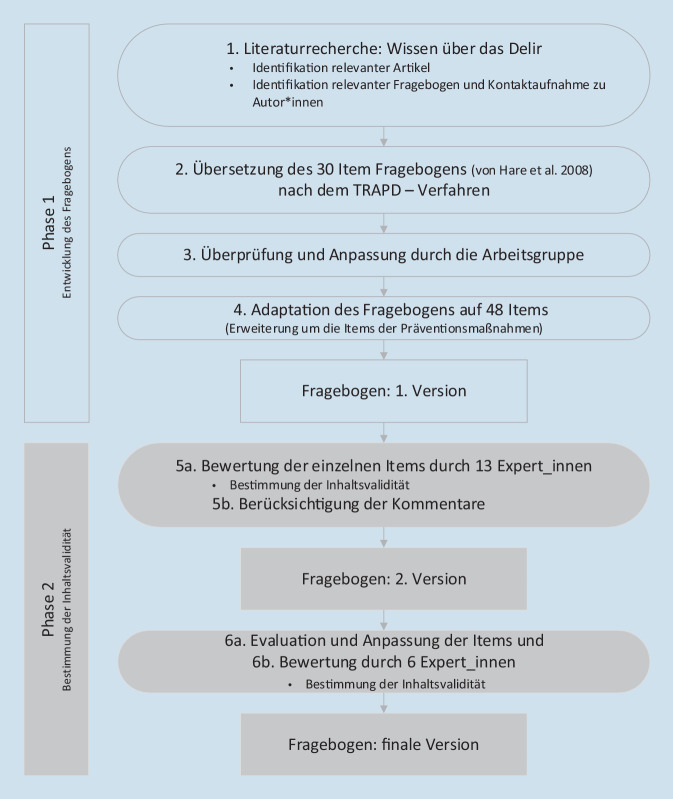

Die Vorgehensweise setzt sich aus 2 Hauptphasen der Fragebogenentwicklung und der Bestimmung der Inhaltsvalidität zusammen (Abb. 1).

## Literaturrecherche, Fragebogenübersetzung und -weiterentwicklung

Zunächst wurde eine Literaturrecherche durch den/die Erstautor_in in den Datenbanken *CINHAL* und *MEDLINE* via PubMed mit den Schlagworten „questionnaire“, „delirium“, „education“ und „knowledge“ durchgeführt. Eingrenzungen erfolgten auf den Zeitraum zwischen 2000 und 2020 sowie auf deutsch- und englischsprachige Artikel. Der Fokus der Recherche lag auf systematischen Übersichtsarbeiten und Studien, die Deliredukationskonzepte entwickelt und evaluiert haben. Zusätzlich wurde in Referenzlisten nach weiteren Artikeln gesucht. Es wurde Kontakt zu mehreren Autor_innengruppen bezüglich der Einsicht in die Fragebögen aufgenommen. Ausgeschlossen wurden Fragebögen, die sich mit der Einstellung, Haltung und dem Selbstvertrauen in das Erkennen und im Management eines Delirs befassten.

Insgesamt wurden im angegebenen Zeitraum nur wenige passende Publikationen identifiziert, die relevante Informationen zum Wissen vom Gesundheitspersonal über das Delir beinhalteten sowie entsprechende Fragebögen entwickelt hatten [8, 13, 20, 30]. Auf Anfrage wurden 2 Fragebögen zur Verfügung gestellt. Der 2008 von Hare et al. [13] entwickelte Fragebogen „A questionnaire to determine nurses’ knowledge of delirium and its risk factors“ wurde ausgewählt, da dieser sowohl Items zum Grundlagenwissen als auch zu relevanten Risikofaktoren enthält. Nach Auskunft der Autor_innen wurde der Fragebogen in 11 Sprachen übersetzt und in 18 Ländern auf mehreren Stationen unterschiedlicher Fachrichtungen eingesetzt. Die Übersetzung ins Deutsche, die Konsentierung und die sprachliche Anpassung erfolgten nach dem Verfahren Translation, Review, Adjudication, Pretest and Documentation (TRAPD) [4, 14]. Das TRAPD-Verfahren ist durch einen Teamansatz gekennzeichnet und durchläuft einen mehrstufigen Prozess. Die Arbeitsgruppe bestand aus der Autor_innengruppe, einer professionellen Übersetzerin, einer weiteren Übersetzerin und einem Moderator.

Der 30 Items umfassende Originalfragebogen beinhaltet die Dimensionen Grundlagenwissen, inklusive Symptome und Management eines Delirs sowie Delirrisikofaktoren. Er wurde vor der Publikation einer Augenscheinvalidität durch Expert_innen unterzogen. Eine Weiterentwicklung erfolgte gemäß internationaler Leitlinien [24, 29] und vorliegender, hochwertiger Übersichtsarbeiten [15, 16, 18] zu nichtpharmakologischen Maßnahmen als zentraler Bestandteil der Delirprävention und des Delirmanagements. Für den erweiterten Fragebogen wurden die Antwortmöglichkeiten „stimme zu“, „stimme nicht zu“ und „unsicher“ aus dem Original beibehalten.

## Auswahl der Expert_innen

Die Auswahl der Delirexpert_innen erfolgte kriteriengeleitet [21, 28]. Sie zeichneten sich durch mindestens eine Publikation zum Thema Delir in einem Peer-Review-Journal oder durch eine ausgewiesene klinische Delirexpertise (z. B. als Pflegeexpert_in – Delirmanagement oder Fachärzt_in für Innere Medizin und Geriatrie) aus. Insgesamt wurden 29 deutschsprachige Expert_innen angefragt. Die Teilnahme war freiwillig und wurde nicht entlohnt. Eine wiederholte Anfrage wurde nach 2 Wochen versendet. Insgesamt belief sich der Beantwortungszeitraum auf 4 Wochen.

## Bestimmung der Inhaltsvalidität anhand des Content Validity Index

Die quantitative Bestimmung der Inhaltsvalidität erfolgte durch die Berechnung des Content Validity Index (CVI). Der CVI gilt als leicht ermittelbares und transparentes statistisches Konstrukt und stellt sich als mehrstufiger Prozess dar [28]. Grundlage des CVI bildete die Bewertung der Relevanz der einzelnen Fragebogen-Items durch Expert_innen [21, 28].

**Figure 2:**
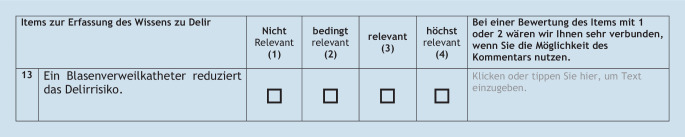

Die Bestimmung des CVI umfasste folgende Schritte: Die Expert_innen schätzten die Relevanz der einzelnen Items auf einem Bewertungsbogen mit einer 4‑stufigen Likert-Skala (1: nicht relevant; 2: bedingt relevant; 3: relevant und 4: höchst relevant) ein (Abb. 2). Danach erfolgte zur Berechnung des CVI gemäß Lynn [21] sowie Polit und Beck [26] die Dichotomisierung in „nicht relevant“ (nicht relevant und bedingt relevant) und „relevant“ (relevant und höchst relevant). Ergänzend war eine Freitextmöglichkeit pro Item gegeben, um Hinweise zur Verständlichkeit oder eine Begründung für das Nichterachten der Relevanz eines Items darzulegen, sowie für sonstige Aspekte [28]. Der Umfang des Fragebogens wurde auf einer 7‑stufigen Likert-Skala (von 1: zu gering, über 4: ausgeglichen, bis 7: zu viel) erfragt, sowie eine Einschätzung zur Zielgruppenempfehlung.

## Datenanalyse

Die Inhaltsvalidierung hat auf Ebene der einzelnen Items (I-CVI-Werte) und der Gesamtskala (S-CVI/Ave) mit vorab definierten Mindestwerten stattgefunden [11, 26–28]. Um aussagekräftige Werte zu generieren, empfehlen Polit et al. [28] 8 bis 12 Expert_innen, Glarcher [11] sogar die Mindestgröße von 13 Expert_innen.

Item-CVI-Werte werden berechnet, indem die Anzahl von relevanten oder höchstrelevanten Bewertungen pro Item durch die Anzahl aller Rater (Expert_innen) dividiert werden. Ab einem Wert ≥ 0,78 gilt ein Item als inhaltsvalide. I‑CVI-Werte, die nur geringfügig kleiner als 0,78 sind, sind unter Einbezug der Kommentare der Expert_innen laut Polit und Beck [27] zu revidieren. I‑CVI-Werte, die deutlich kleiner als 0,78 sind, werden verworfen. Spezifische Grenzwerte wurden von den Autor_innen nicht vorgegeben. Items, die trotz gutem I‑CVI anhand der Kommentare der Expert_innen durch mangelnde Verständlichkeit auffallen, können sprachlich und inhaltlich angepasst werden. Um die Inhaltsvalidität auf Gesamtskalenlevel (S-CVI/Ave) zu berechnen, werden alle I‑CVI-Werte addiert und durch die Anzahl der Items dividiert. Der S‑CVI/Ave-Wert steht ab 0,9 für eine gute bis exzellente Inhaltsvalidität [21, 26–28].

In der zweiten Runde wurde der überarbeitete Fragebogen, wie von Lynn [21] sowie Polit und Beck [27] empfohlen, mit einer kleineren Expert_innengruppe bewertet. Dabei gilt, dass bei weniger als 5 Expert_innen der I‑CVI immer 1,00 ergeben muss. Bei 5 bis 8 Expert_innen kann eine nichtgegebene Zustimmung toleriert werden. Damit das Ergebnis um zufällige Übereinstimmungen korrigiert und an die jeweilige Zustimmung zur inhaltlichen Relevanz adaptiert werden konnte, wurde der modifizierte Kappa (κ*) nach Polit et al. [28] berechnet. Somit wurde auch der Grad der Übereinstimmung unter den Expert_innen bei der Itembewertung miteinbezogen. Um die Methodenkritik von Beckstead (2009) [3] zu berücksichtigen, wurde zusätzlich die untere Grenze des 95 %igen Konfidenzintervalls (KI) von κ* berechnet.

Die statistischen Auswertungen erfolgten mit „SPSS Statistics 27“ (IBM, Endicott, NY, USA) und „Excel 2016“ (Microsoft, Redmond, WA, USA).

## Ergebnisse

### Expert_innenrunde

An der ersten Bewertungsrunde des Fragebogens beteiligten sich 13 von 29 Expert_innen, 9 Pflegefachpersonen aus Forschung und Praxis und 4 Mediziner_innen. Die berufliche Qualifikation der Pflegefachpersonen reichte von der klassischen Pflegeausbildung mit mehrjähriger Berufserfahrung und/oder einer vertiefenden Fachausbildung (z. B. Fachweiterbildung oder Studium der Gerontologie, Advanced Practice Nursing, Pflegepädagogik) bis zur Habilitation im Bereich der Pflegewissenschaft. Die Mediziner_innen haben promoviert und teilweise eine Professur inne. Die zweite Bewertungsrunde bestand aus 5 Expert_innen der Pflege und einem Mediziner mit Facharztausbildung im Bereich der Psychiatrie.

### Inhaltsvalidierung

#### Bewertungsrunde

**Figure 3:**
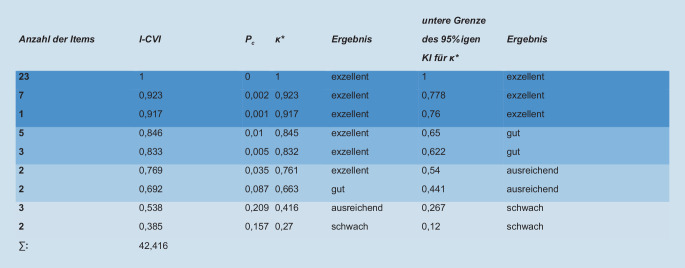

Von den 48 Fragebogenitems sind aufgrund der Bewertung der Expert_innen 6 entfernt, 12 revidiert und 30 Items belassen worden. Die I‑CVI- und modifizierten Kappa(κ*)-Werte sowie die Untergrenze des 95 %igen Konfidenzintervalls (KI) sind in Abb. 3 zusammengefasst.

Im Zusatzmaterial Online Supplement 1 sind die Iteminhalte des Fragebogens und im Zusatzmaterial Online Supplement 2a die detaillierte Ergebnisbeschreibung der 1. Bewertungsrunde dargestellt (siehe auch [1, 9, 12]).

Der Gesamtskalenwert S‑CVI/Ave erreichte in der ersten Bewertungsrunde einen Wert von 0,884. Den Umfang des Fragebogens bewerteten die Expert_innen mit einem Mittelwert von 4,9 bzw. einem Median von 5 als eher „viel“.

Bei der Frage, für welche Zielgruppe der Fragebogen geeignet ist, gab es unterschiedliche Ansichten. Der Anwendbarkeit für Pflegefachpersonen stimmten alle Expert_innen zu, für Therapeut_innen 11 Expert_innen. Dass er für Mediziner_innen geeignet sei, gaben 9 von 13 Expert_innen an, von den 4 Mediziner_innen 3. Für An- und Zugehörige sowie Hilfspersonal ist der Fragebogen nicht geeignet, da er gemäß der Expertenrückmeldung nicht niederschwellig konzipiert worden ist.

Im letzten Teil des Fragebogens wurden von den Expert_innen sowohl formale als auch inhaltliche Hinweise gegeben. Die Redundanz einiger Fragen, die teilweise fehlende Lesefreundlichkeit durch Verneinungsfragen sowie der Wunsch nach weiteren Inhalten wurden angemerkt. Items zu expliziten Screeninginstrumenten, auch in Abgrenzung zu Assessmentinstrumenten, nach delirogenen Medikamenten, zu weiteren Delirsymptomen und zur Abgrenzung Delir/Demenz wurden gewünscht. Für den ärztlichen Dienst wurde beispielsweise der adäquate Einsatz von Medikamenten als fachspezifischer Inhalt empfohlen.

Nach der Überarbeitung des Fragebogens verblieben 42 Items.

#### Bewertungsrunde

Für die zweite Bewertungsrunde wurden 6 Expert_innen aus der ersten Kohorte kontaktiert, die gemäß der Empfehlung von Polit et al. [28] konstruktive Hinweise gegeben hatten. Eine Rückmeldung konnte nicht in die quantitative Auswertung miteinbezogen werden, da es in der Plausibilitätsprüfung Unklarheiten gab.

Fünf Expert_innen erachteten 41 von 42 Items des überarbeiteten Fragebogens als relevant. Der I‑CVI ergab für 41 Items eine exzellente Übereinstimmung (I-CVI 1,0). Die Ergebnisbeschreibung befindet sich im Zusatzmaterial Online Supplement 2b.

In der finalen Version des Fragebogens verblieben somit 41 Items; der Gesamtskalenwert (S-CVI/Ave) zeigt mit 1,0 eine exzellente Inhaltsvalidität. Wichtige didaktische Impulse in den Kommentaren wurden berücksichtigt und 2 Formulierungen adaptiert (Item 28, 44).

Während der Umfang des Fragebogens mit 4,0 vom Median her ausgeglichen war, wurde er mit einem Mittelwert von 4,6 bewertet.

Die Rückmeldungen zur Zielgruppen änderten sich im Vergleich zur ersten Bewertungsrunde nicht. Der finale Fragebogen befindet sich als Zusatzmaterial Online Supplement 3 im Anhang.

## Diskussion

Ziel dieser Studie war es, einen deutschsprachigen Fragebogen des Wissens über das Delir zu entwickeln und diesen inhaltlich zu validieren. Zur Prüfung der Inhaltsvalidität wurde der im pflegewissenschaftlichen Setting breit akzeptierte CVI angewendet. Der Originalfragebogen enthielt Fragen zu Grundlagenwissen und Risikofaktoren eines Delirs. Da erwiesen ist, dass durch die Anwendung nichtpharmakologischer Präventionsmaßnahmen die Versorgungsqualität erhöht und bis zu 40 % der Delire [15, 16, 18] potenziell vermieden können, erhält die Weiterentwicklung des nun vorliegenden Fragebogens einen Mehrwert. Wie im internationalen Forschungskontext nachgewiesen werden konnte, ist das Wissen bei pflegerischem und ärztlichem Personal oft unzureichend [5, 7, 8, 13, 17, 19]. Der hier entwickelte Fragebogen zeigte sich als inhaltsvalide. Damit ist es möglich, den Wissenstand zum Delir zu erfassen.

Der Umfang des finalen Fragebogens wurde durch die Reduktion der Items im Median als „ausgeglichen“ eingestuft. Weiteres Kürzungspotenzial wurde in redundanten Iteminhalten gesehen. Da diese laut Polit und Beck [27] durchaus gewollt sind, wurden sie beibehalten.

Die Adressierung der Zielgruppe zeigte sich in beiden Bewertungsrunden deckungsgleich. Grundsätzlich ist der Fragebogen zum Wissensstand für verschiedene Berufsgruppen geeignet. Für Pflegefachpersonen und Therapeut_innen gab es die meiste Zustimmung. Da auch 3 von 4 Mediziner_innen den Fragebogen als potenziell geeignet für Mediziner_innen hielten, wäre ein Einsatz grundsätzlich mit zusätzlichen tiefergehenden Inhalten denkbar.

Trotz der breiten Akzeptanz des CVI als Methode zur Bestimmung der Inhaltsvalidität, finden sich in der Literatur kontroverse Diskurse. Ein Hauptkritikpunkt adressiert die Überschätzung der wahren Übereinstimmung durch eine mögliche Zufallsübereinstimmung zwischen den Expert_innen. Um dem zu begegnen, wurde, wie von Beckstead (2009) und Glarcher [11] empfohlen, auch die Untergrenze des 95 %igen KI des κ* berechnet. Unsere Ergebnisse bestätigen die Kritik. Waren einige Items bei der Übereinstimmung nach ihrer Relevanz zunächst als exzellent, gut oder ausreichend nach Berechnung des κ* bewertet, stellten sich diese durch die Berücksichtigung der Untergrenze des 95 %-igen KI teilweise nur noch als gut, ausreichend oder schwach dar.

Die statistische Wahrscheinlichkeit für das Auftreten von „relevanten“ und „nicht relevanten“ Bewertungen sinkt laut Beckstead (2009) stark mit einer kleinen Gruppe. Glarcher [11] empfiehlt zur Erreichung aussagekräftiger CVI-Werte eine Größe der Gruppe von mindestens 13 anstelle von 8 bis 12 Expert_innen, wie von Polit et al. [28] gefordert. Um den Empfehlungen Rechnung zu tragen, haben wir in der ersten Bewertungsrunde 13 Expert_innen gewinnen können. In der zweiten Bewertungsrunde wurde aufgrund der herausfordernden Rekrutierung während der SARS-CoV-2-Pandemie darauf verzichtet, nochmals alle 13 Expert_innen um Unterstützung zu bitten. Somit konnte der Kritik von Glarcher [11] und Beckstead [3] nicht vollständig begegnet werden. Anstelle dessen wurde, wie von Lynn [21] und Polit et al. [27] empfohlen, der überarbeitete Fragebogen in einer kleineren Gruppe ein zweites Mal bewertet. Möglicherweise sind die erzielten Ergebnisse des CVI, trotz zusätzlicher Berücksichtigung von κ* und der Untergrenze des 95 %igen KI, somit nicht vollumfänglich verlässlich.

Kritik aus pädagogischer Sicht betraf v. a. anwenderunfreundliche Aspekte wie die eingeschränkte Lesefreundlichkeit einiger Fragestellungen, redundante Inhalte und Verneinungsfragen. Letztere konnten aufgrund der Konstruktion eines nominal skalierten Fragebogens mit geschlossenen Fragen und den vorgegebenen Antwortmöglichkeiten nicht gänzlich vermieden werden. Laut Polit und Beck [27] sind positiv formulierte Items zu bevorzugen, aber Verneinungsfragen durchaus möglich, um einer möglichen Zustimmungstendenz entgegenzuwirken. Polit und Beck [27] empfehlen weiterhin, auf Verallgemeinerungen bei der Formulierung zu verzichten. Dem wurde Rechnung getragen, indem Wörter wie „immer“ und „nie“ abgeschwächt wurden.

## Limitationen

Aufgrund eingeschränkter Ressourcen war keine systematische Literaturrecherche möglich, sodass publizierte Fragebogen übersehen worden sein könnten. Dem Wunsch nach Integration weiterer Themeninhalte konnte aufgrund der kleineren Gruppengröße der Expert_innen in der zweiten Bewertungsrunde nicht nachgegangen werden. Dieser Impuls sollte Eingang in die Entwicklung weiterer Dimensionen, wie z. B. Haltung, Einstellung und Selbstvertrauen in das eigene Handeln finden. Die Bewertung der Relevanz der Items durch Expert_innen geht grundsätzlich mit einer gewissen Subjektivität einher.

## Schlussfolgerung/Ausblick

Die Bestimmung der Inhaltsvalidität mittels CVI hat sich als geeignete Methode erwiesen, wenn mindestens 13 Expert_innen befragt und die Untergrenze des 95 %igen KI des κ* berechnet wird. Der vorliegende Fragebogen zeigt nach 2 Bewertungsrunden eine sehr gute Inhaltsvalidität. Er ist geeignet, um aktuelles, evidenzbasiertes Wissen der Pflegefachpersonen über das Delir einschätzen zu können, z. B. als Status-quo-Erhebung bei Fortbildungen, zur Selbstevaluation der Lernenden oder als Lernerfolgskontrolle etc. Vorstellbar wäre darüber hinaus der Einsatz im Kontext eines interprofessionellen Edukationsprogramms. Die Auswertung kann über die Anzahl an richtig beantworteten Fragen, wie im Originalfragebogen, erfolgen. Die Ergebnisbewertung (erreichte Punktzahl) sollte dabei mit der didaktischen Zielsetzung und der jeweiligen Zielgruppe im Einklang stehen.

## Fazit für die Praxis

- Erstmals liegt ein inhaltsvalider Fragebogen zur Erfassung des Wissens zum Delir in deutscher Sprache vor.
- Er ist geeignet, das Wissen über das Delir zu evaluieren und zielgerichtet Schulungen zu konzipieren.
- Der Fragebogen wird primär für Pflegefachpersonen empfohlen und ist mit einer Anpassung auch für therapeutisches und ärztliches Personal denkbar.

## Supplementary Information






