# Supplementary material for: Development and content validity of a questionnaire to assess knowledge about delirium
Source: Z Gerontol Geriatr. 2022 Jan 26;56(2):132–8. [Article in German] doi: 10.1007/s00391-022-02015-9 (PMC8791090; doi:10.1007/s00391-022-02015-9)

**Online Supplement 1: Beschreibung der Fragebogenitems 1. Version**

| Itemnummer | Dimension | Fragestellung zu |
| --- | --- | --- |
| 1 | Grundlagenwissen | Delirsymptomatik – Symptomkomplex |
| 2 |  | Screening- und Assessmentinstrumente |
| 3 |  | Delirsymptom – Orientierung |
| 4 |  | Hypoaktives Delir – Depression |
| 5 |  | Delirbehandlung – Sedierung |
| 6 |  | Delirverlauf |
| 7 |  | Instrument zur Diagnosestellung |
| 8 | Risikofaktor | Delirursache – Operation |
| 9 | Grundlagenwissen | Delirverlauf – Dauer |
| 10 | Risikofaktor | Alter |
| 11 |  | Sensorische Beeinträchtigung |
| 12 |  | Medikation |
| 13 |  | Blasenkatheter |
| 14 |  | Geschlecht |
| 15 |  | Ernährung |
| 16 |  | Demenz |
| 17 |  | Geschlecht |
| 18 |  | Diabetes |
| 19 |  | Flüssigkeit |
| 20 |  | Sensorische Beeinträchtigung |
| 21 |  | Übergewicht |
| 22 | Grundlagenwissen | Delirsymptom – Teilnahmslosigkeit |
| 23 |  | Delirsymptom – Aggressivität |
| 24 |  | Delirursache – Alkoholentzug |
| 25 |  | Delirfolge – erhöhte Sterblichkeit |
| 26 | Risikofaktor | Demenz |
| 27 | Grundlagenwissen | Delirsymptom – Fluktuation |
| 28 |  | Delirsymptom – Aufmerksamkeitsstörung |
| 29 |  | Delirsymptom – Wahrnehmungsstörungen |
| 30 |  | Delirsymptom – Tag-Nacht-Rhythmus |
| 31 | Präventionsmaßnahmen | Sensorische Beeinträchtigung |
| 32 |  | Maßnahmenbündel |
| 33 |  | Kognitive Aktivierung |
| 34 |  | Flüssigkeit |
| 35 |  | Mobilität |
| 36 | Grundlagenwissen | Freiheitseinschränkende Maßnahmen |
| 37 | Präventionsmaßnahmen | Infektion |
| 38 |  | Infektion |
| 39 |  | Mobilität |
| 40 |  | Orientierung |
| 41 | Risikofaktor | Schmerzen |
| 42 | Präventionsmaßnahmen | Medikation |
| 43 |  | Mobilität |
| 44 |  | An- und Zugehörige |
| 45 |  | Atmung |
| 46 |  | Ernährung |
| 47 |  | Ausscheidung |
| 48 |  | Tag-Nacht-Rhythmus |


Supplement: Supplementary file 1 [file 391_2022_2015_MOESM1_ESM.docx]
