# Supplementary material for: Development and content validity of a questionnaire to assess knowledge about delirium
Source: Z Gerontol Geriatr. 2022 Jan 26;56(2):132–8. [Article in German] doi: 10.1007/s00391-022-02015-9 (PMC8791090; doi:10.1007/s00391-022-02015-9)
Supplement: Supplementary file 2 [file 391_2022_2015_MOESM2_ESM.docx]

**Online Supplement 2a und 2b**

**Detaillierte Ergebnisbeschreibung der 1. und 2. Bewertungsrunde**

*2a: 1.Bewertungsrunde*

Durch die Bewertung der Expert_innen zeigten 39 der 48 Fragebogenitems mit I-CVI-Werten ≥0,78 eine gute Inhaltsvalidität. Der *k**, in der Interpretation nach Fleiss [9], zeigte für 41 Items (Range von 0,76-1) exzellente Werte. Von diesen 41 Items erreichten 31 in der Untergrenze des 95%-igen KI exzellente Werte. Zwei Items (4%) aus dem ursprünglichen Fragebogen wurden aufgrund ihres I-CVI von 0,38 und unzureichenden k*-Werten auf beiden Ebenen verworfen. Es gibt nach neuesten Erkenntnissen keine wissenschaftlichen Belege dafür, dass Männer (Item17) ein höheres Delirrisiko haben [1, 12]. Der Risikofaktor Übergewicht (Item 21) wurde in den Kommentaren als nicht „nah genug am Phänomen“ erachtet. Sieben Items (14,6%) erreichten moderate I-CVI-Werte von 0,53-0,77. Nach Kalkulation auf den beiden Ebenen des *k** erreichten die Items jedoch nur ausreichende und schwache Ergebnisse und wurden aufgrund dessen revidiert. Unter Berücksichtigung der Kommentare wurden die Items (Item 2, 8) überarbeitet und bei Dopplungen (Item 7, 35) beziehungsweise uneinheitlicher wissenschaftlicher Belege der Items (Item 14, 18) verworfen. Diese Erkenntnisse basierten auf einer aktuellen Literaturrecherche [1, 12] durch die Autor_innen und aufgrund der Rückmeldung der Expert_innen. Item 45 wurde aufgrund seines annähernd guten Wertes von 0,77 und ohne Kommentare der Rater beibehalten. Die Frage nach Demenz in der Familienanamnese als Delir-Risikofaktor, wurde nach substanzieller Kritik mehrerer Expert_innen in eine Abgrenzungsfrage zu Delir/ Demenz umformuliert (Item 26). Obwohl einige der Items mit I-CVI-Werten ≥0,78 eine gute Inhaltsvalidität aufwiesen, wurden aufgrund der Rückmeldungen der Expert_innen bei mehreren Items sprachliche Anpassungen zur besseren Verständlichkeit vorgenommen. Um Verallgemeinerungen zu umgehen, wurden Worte wie „nie“ und „immer“ (Item 6, 8, 9, 23) abgeschwächt oder verändert. Auf der inhaltlichen Ebene wurden fünf Items nach der Auswertung der Kommentare leicht modifiziert (Item 2, 4, 16, 32, 42). Beispielsweise wurde das Wort „hypoaktiv“ hinzugefügt (Item 4) oder die Tabelle mit den Bewertungsinstrumenten adaptiert (Item 2).

*2b: 2.Bewertungsrunde*

Item 45 (Überwachung der Sauerstoffsättigung) wurde einmal als nicht relevant eingeschätzt. Es erfüllte mit einem I-CVI-Wert von 0,83 die Mindestanforderung, war aber nach der Adaption an die Untergrenze des 95%-igen KIs nur ausreichend und wurde entfernt.
